# Blood groups and Rhesus status as potential predictors of outcomes in patients with cardiac resynchronisation therapy

**DOI:** 10.1038/s41598-024-58747-8

**Published:** 2024-04-10

**Authors:** Nikolaos Papageorgiou, Catrin Sohrabi, Constantinos Bakogiannis, Anastasios Tsarouchas, Kishore Kukendrarajah, Luso Matiti, Neil T. Srinivasan, Syed Ahsan, Simon Sporton, Richard J. Schilling, Ross J. Hunter, Amal Muthumala, Antonio Creta, Anthony W. Chow, Rui Providencia

**Affiliations:** 1 Electrophysiology Department, Barts Heart Centre, St. Bartholomew’s Hospital, West Smithfield, London, UK; 2 Institute of Cardiovascular Science, University College London, London, UK; 3 3rd Cardiology Department, AUTH, Ippokrateio Hospital, Thessaloniki, Greece; 4 The Farr Institute of Health Informatics Research, University College London, London, UK; 5 Broomfield Hospital, Mid and South Essex NHS Foundation Trust, Essex, UK; 6 Department of Cardiac Electrophysiology, Essex Cardiothoracic Centre, Basildon, UK; 7 Circulatory Health Research Group, Medical Technology Research Centre, School of Medicine, Anglia Ruskin University, Chelmsford, UK

**Keywords:** Blood groups, Heart failure, Cardiac resynchronisation therapy, Cardiac device therapy, Cardiovascular biology

## Abstract

Cardiac resynchronisation therapy (CRT) improves prognosis in patients with heart failure (HF) however the role of ABO blood groups and Rhesus factor are poorly understood. We hypothesise that blood groups may influence clinical and survival outcomes in HF patients undergoing CRT. A total of 499 patients with HF who fulfilled the criteria for CRT implantation were included. Primary outcome of all-cause mortality and/or heart transplant/left ventricular assist device was assessed over a median follow-up of 4.6 years (IQR 2.3–7.5). Online repositories were searched to provide biological context to the identified associations. Patients were divided into blood (O, A, B, and AB) and Rhesus factor (Rh-positive and Rh-negative) groups. Mean patient age was 66.4 ± 12.8 years with a left ventricular ejection fraction of 29 ± 11%. There were no baseline differences in age, gender, and cardioprotective medication. In a Cox proportional hazard multivariate model, only Rh-negative blood group was associated with a significant survival benefit (HR 0.68 [0.47–0.98], p = 0.040). No association was observed for the ABO blood group (HR 0.97 [0.76–1.23], p = 0.778). No significant interaction was observed with prevention, disease aetiology, and presence of defibrillator. Rhesus-related genes were associated with erythrocyte and platelet function, and cholesterol and glycated haemoglobin levels. Four drugs under development targeting RHD were identified (Rozrolimupab, Roledumab, Atorolimumab, and Morolimumab). Rhesus blood type was associated with better survival in HF patients with CRT. Further research into Rhesus-associated pathways and related drugs, namely whether there is a cardiac signal, is required.

## Introduction

Heart failure (HF) is characterised by symptomatic left ventricular dysfunction due to structural or functional impairment of the ventricles. At present, HF remains a growing public health concern and economic burden affecting over 60 million individuals worldwide^1^. In the United States, HF is associated with a 1-year mortality rate of approximately 20–25%, over 1 million hospitalisations per year, and an annual expenditure exceeding $30 billion^2,3^.

The syndrome of HF overlaps with other cardiovascular conditions and the underlying aetiology can be diverse. In clinical practice, treatment includes preventative strategies targeting well-established risk factors in addition to the use of multiple drug classes in patients with established HF e.g., beta-blockers, angiotensin-converting enzyme inhibitors, mineralocorticoid receptor antagonists, neprilysin inhibitors and, more recently, sodium-glucose co-transporter 2 inhibitors. In addition to pharmacological therapy, devices including implantable cardiac defibrillators and cardiac resynchronisation therapy (CRT) play a key role in the management of HF. However, prognosis remains poor and approximately one third of patients do not respond or have a suboptimal response to management^4,5^. Overall, CRT response is poorly defined and may be characterised by mechanical, echocardiographic, and physiological abnormalities. Numerous studies have identified predictors of this, highlighting the importance of clinical risk evaluation^6–9^.

Knowledge of the pathways that underpin HF are incompletely understood but have the potential to inform novel therapeutic strategies, guide future risk stratification, and to predict clinical response to therapy. Although over 140 genomic loci have been identified via genome-wide association studies (GWAS) for conditions such as atrial fibrillation^10^, only 11 loci have been linked to HF^11^. More recently, a phenome-wide scan using data from the UK Biobank identified an association between ABO blood group and myocardial infarction, thromboembolism, and HF, with a higher risk for non-O blood group patients^12^. Moreover, the ABO gene locus has been shown to influence angiotensin-converting enzyme activity^13^ and result in increased circulating levels of atherosclerosis-associated endothelial adhesion molecules^14^. The non-O blood group gene also encodes a protein with glycotransferase activity that modifies von Willebrand factor resulting in impaired proteolysis with increased risk of thrombosis^15^, and the present of a non-O blood group is associated with increased cholesterol levels^16^. A genetic link has also been found between Rhesus status and cardiovascular disease^17–20^. Whether these factors are associated with survival outcomes in HF patients managed with CRT has yet to be determined and understanding their importance may help guide clinical decision making and optimise outcomes for patients. In this study, we aimed to evaluate the significance of blood groups in HF patients managed with CRT.

## Methods

This was a retrospective, single-center observational study involving consecutive HF patients who underwent successful implantation of a CRT-P/D device preceded by routine blood grouping at the Heart Hospital/University College London Hospital and Barts Heart Centre UK between May 2000 and March 2015. Auditing outcomes and complications post-CRT implantation was approved locally via the Barts Clinical Effectiveness Unit (Project ID: 11056) and on a National level as part of the National Institute for Cardiovascular Outcomes Research (NICOR) Device Audit (approved by the National Information Governance Board for Health and Social Care, with section 251 approval as part of the NHS Act 2006)^21^. All methods were performed in accordance with the relevant guidelines and regulations. Implantation of CRT is a part of routine clinical practice, and no experiments were involved in this study. All patients provided written and informed consent prior to the procedure. The data were collected retrospectively through hospital electronic records and additional information was retrieved from paper notes where needed. Local clinic records and stored device electrograms were also used for data collection.

The following inclusion criteria were used for patients receiving CRT: documented HF of New York Heart Association (NYHA) class II–IV, symptoms despite optimal drug therapy, LV ejection fraction (LVEF) ≤ 35%, and QRS duration ≥ 120 ms, in line with European Society of Cardiology (ESC) guidelines^22^. The choice of pacemaker or defibrillator was based on clinical history, risk profile, and history of arrhythmia. Exclusion criteria included patients requiring intravenous inotropic drug therapy or having an estimated life expectancy of less than 12 months due to comorbidities other than HF.

Blood samples were collected on the day of the procedure or during the pre-assessment visit (less than 5 days before the procedure). These were used to determine the blood group of each patient in preparation for the procedure, and an institutional protocol was used to deal with procedure-related major bleeds. Patients implanted with CRTs, during the 2000s were considered high procedural risk due to the presence of heart failure with reduced ejection fraction and concerns regarding potential bleeding complications (e.g., due to perforation of the coronary sinus or right ventricle). Accordingly, blood grouping was routinely performed at our institutions during the pre-assessment visit for elective patients, or prior to procedure in other cases. Currently, blood grouping in our institution for CRT implants is only performed for very specific cases of patients considered high bleeding risk.

In total, 499 patients were followed-up for a median of 4.6 years (IQR 2.3–7.5) post-CRT implantation. More than 10% of patients had follow-up for ≥ 10 years. The primary study endpoint was a composite of all-cause mortality and/or heart transplantation/LV assistant device (LVAD).

### Statistical analysis

Data are presented as mean ± standard deviation (SD) for continuous variables and as valid percentages for categorical variables. Sub-analyses were conducted for device indication (primary vs. secondary prevention), device type (CRT-P vs. CRT-D), and underlying HF aetiology (ischemic vs. non-ischemic).

Continuous variables were tested for normality of distribution with the Kolmogorov–Smirnov test and by visual inspection of P–P plots. To examine the differences in demographic and clinical characteristics according to ABO and Rh blood types, one-way analysis of variance (ANOVA) and X^2^ tests were used for continuous and categorical variables. Based on previous studies^12,23,24^, patients were grouped as O-type or non-O type (including A, B, and AB types), and Rh-positive or Rh-negative. Multivariate Cox regression analysis was used to test the time-dependency of association of blood groups and the study endpoints. Data was censored at date of available follow-up or when patients reached the endpoint of interest. Adjustment for baseline differences was performed as required (Method: Forward LR, probability for stepwise < 0.05). Two further multivariate models were tested to assess robustness of findings (Method: Enter and Backward LR, probability for stepwise < 1.0). Exact values of p < 0.05 were considered statistically significant. Data analysis was performed with SPSS software, version 18.0 (SPSS Inc., Chicago, IL).

The human RH locus consists of two structural genes, RHD (coding the D antigen) and RHCE (CcEe antigens), which have been mapped to the short arm p34–36 of chromosome 1^25^. Expression of A or B antigens, results from allelic combinations of genetic variants in the ABO gene, located on chromosome 9 (9q34.2)^12^.

For the identified associations of blood groups with outcomes we searched the NHGRI-EBI Catalog of human genome-wide association studies (GWAS-Catalog) for previously described associations of the culprit gene with clinical traits^26^. Subsequently, we searched the Drug Gene Interaction Database (DGIdb)^27^, Open Targets^28^, and ChEMBL^29^ for druggabilty status and any available drugs targeting those genes.

## Results

### Demographic characteristics

Demographic characteristics are presented in Table 1. A total of 499 patients were included in this study. Most patients had an O-type (n = 226; 45.3%) and Rh-positive blood group (n = 423; 84.8%). The average age of all patients was 66 ± 12.8 years and 28.5% (n = 142) of patients were female. There was no significant difference in the prevalence of diabetes mellitus, atrial fibrillation, chronic obstructive pulmonary disease (COPD), or stroke between groups. In addition, BMI was similar between groups as was NYHA classification; however, LVEF was higher in Rh-negative vs. Rh-positive patients (p = 0.045). In this cohort, 85.0% (n = 424) received a CRT with defibrillator for primary prevention of sudden cardiac death and 30.7% (n = 153) received CRT upgrade. Overall, there was no difference in the use of cardioprotective medication between groups.

**Table 1:** Baseline demographic characteristics.

Variable	Total (n = 499)	Rhesus (Rh) blood group		ABO blood group			
		Rh− (n = 76)	Rh + (n = 423)	O (n = 226)	A (n = 185)	B (n = 61)	AB (n = 27)
Age	66.4 ± 12.8	66.5 ± 12.8	66.4 ± 12.8	67.1 ± 12.4	66.5 ± 13.2	65.1 ± 12.5	63.2 ± 13.9
Women	28.5% (142)	21.1% (16)	29.8% (126)	30.1% (68)	24.3% (45)	34.4% (21)	29.6% (8)
BMI	28.2 ± 5.9	28.1 ± 5.9	28.2 ± 5.9	28.2 ± 5.8	28.0 ± 5.6	28.5 ± 6.6	29.2 ± 7.9
Diabetes mellitus	26.5% (132)	28.9% (22)	26.1% (110)	26.5% (60)	22.3% (41)	32.8% (20)	40.7% (11)
Atrial Fibrillation	42.7% (212)	51.3% (39)	41.2% (173)	44.7% (101)	41.8% (76)	37.7% (23)	44.4% (12)
COPD	9.4% (47)	7.9% (6)	9.7% (41)	10.2% (23)	8.2% (15)	9.8% (6)	11.1% (3)
Stroke	7.2% (36)	2.6% (2)	8.1% (34)	7.1% (16)	7.1% (13)	9.8% (6)	3.7% (1)
Ischaemic CM	48.3% (241)	48.7% (76)	48.2% 423)	47.3% (107)	49.7% (92)	44.3% (27)	55.6% (15)
CRT-P	22.4% (112)	23.7% (18)	22.2% (94)	25.2% (57)	19.5% (36)	24.6% (15)	14.8% (4)
Primary prevention device	85.0% (424)	84.2% (64)	85.1% (360)	84.1% (190)	84.3% (156)	91.8% (56)	81.5% (22)
CRT upgrade	30.7% (153)	27.6% (21)	31.3% (132)	32.3% (73)	27.7% (51)	31.1% (19)	37.0% (10)
NYHA	2.6 ± 0.7	2.6 ± 0.7	2.6 ± 0.7	2.7 ± 0.7	2.6 ± 0.7	2.7 ± 0.6	2.7 ± 0.7
LVEF	29 ± 11	31 ± 10*	29 ± 11	29 ± 11	30 ± 11	27 ± 9	30 ± 8
QRS baseline	163 ± 24	162 ± 25	163 ± 24	165 ± 24	161 ± 25	164 ± 26	161 ± 25
eGFR	58 ± 22	59 ± 19	58 ± 23	58 ± 21	57 ± 21	25 ± 25	68 ± 26
Beta-blocker	65.2% (324)	59.2% (45)	66.3% (279)	64.6% (146)	66.1% (121)	67.2% (41)	59.3% (16)
ACEi/ARB-II	87.3% (433)	89.5% (68)	86.95 (365)	87.1% (196)	88.0% (161)	83.6% (51)	92.6% (25)
MRA	55.4% (275)	52.6% (40)	56.0% (235)	57.3% (129)	49.7% (91)	62.3% (38)	63.0% (17)
Oral anticoagulants	43.1% (214)	50.0% (38)	41.8% (176)	44.7% (101)	42.1% (77)	42.6% (26)	37.0% (10)
Antiplatelets	47.8% (237)	39.5% (30)	49.3% (420)	44.9% (101)	49.2% (90)	55.7% (34)	44.2% (12)

*P = 0.045; All other comparisons P = NS.

### Predictors of all-cause mortality, heart transplant, and left ventricular assist device

At follow-up, 262 (52.5%) patients died and 14 (2.8%) received transplantation or were fitted with an LVAD. Our analysis showed that age, female gender, BMI, ischaemic CM, LVEF, QRS ≥ 150 ms, NYHA class, atrial fibrillation, COPD, previous stroke, eGFR ≥ 60 mL/min, and Rh-negative blood group were associated with all-cause mortality and/or heart transplant/LVAD (Table 2). On multivariate analysis, we found that age (HR 1.02 [1.00–1.03], p = 0.010) was an independent predictor. In addition, female gender (HR 0.74 [0.56–0.98], p = 0.038), LVEF (HR 0.98 [0.97–0.99], p < 0.001), QRS ≥ 150 ms (HR 0.51 [0.40–0.66], p < 0.001), eGFR ≥ 60 mL/min (HR 0.62 [0.47–0.82], p < 0.001), and Rh-negative blood group (HR 0.68 [0.47–0.98], p = 0.040) were independent predictors of patient outcome and significantly associated with reduced all-cause mortality and/or heart transplant/LVAD. The role of Rh-negative blood group as an independent predictor of survival was further elucidated using two additional Multivariate Cox regression models (Supplementary Table 1).

**Table 2:** Predictors of all-cause mortality and/or heart transplant/LVAD.

Predictor	Univariate Cox regression			Multivariate Cox regression (forward LR)		
	HR	95%CI	P	HR	95%CI	P
Age (per year)	1.03	1.01–1.04	< 0.001	1.02	1.00–1.03	0.010
Women	0.70	0.53–0.92	0.011	0.74	0.56–0.98	0.038
BMI (per Kg/m^2^)	0.97	0.95–0.99	0.009	–	–	–
ICD	1.14	0.85–1.52	0.376	–	–	–
Secondary prevention	0.95	0.68–1.33	0.765	–	–	–
Ischaemic CM	1.53	1.21–1.94	< 0.001	–	–	–
LVEF (%)	0.98	0.96–0.99	< 0.001	0.98	0.97–0.99	< 0.001
QRS ≥ 150 ms	0.59	0.46–0.76	< 0.001	0.51	0.40–0.66	< 0.001
NYHA class (per class)	2.02	1.65–2.47	< 0.001	–	–	–
AF	1.27	1.00–1.61	0.049	1.24	0.97–1.58	0.084
Diabetes mellitus	1.18	0.91–1.54	0.214	–	–	–
COPD	1.51	1.05–2.18	0.026	–	–	–
Previous stroke	1.51	1.01–2.25	0.043	–	–	–
eGFR ≥ 60 mL/min	0.54	0.42–0.69	< 0.001	0.62	0.47–0.82	< 0.001
Rhesus neg (−)	0.67	0.47–0.96	0.029	0.68	0.47–0.98	0.040
Non-O group	0.97	0.76–1.23	0.778	–	–	–

**Figure 1:**
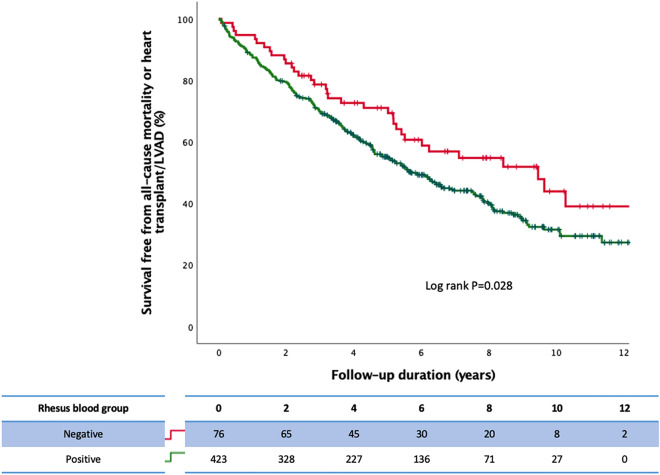
Survival free from all-cause mortality or heart transplant/LVAD in HF patients with CRT and either Rh-negative or Rh-positive status.

**Figure 2:**
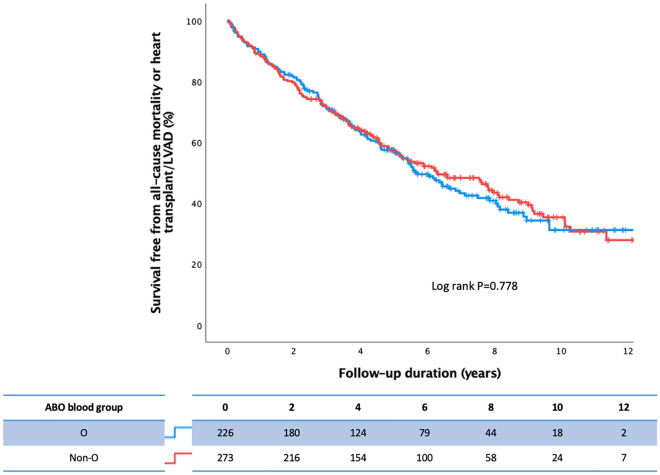
Survival free from all-cause mortality or heart transplant/LVAD in HF patients with CRT and either O or non-O type blood group.

Survival free from all-cause mortality or heart transplant/LVAD is shown in Fig. 1. Rh-negative patients demonstrated significantly greater survival when compared with Rh-positive patients. No significant difference was observed in patients with O-type vs. non-O type (including A, B, and AB types) blood group with respect to the primary outcome (Fig. 2).

### Exploratory sub-analyses for effect of Rhesus status by disease aetiology (ischaemic vs. non-ischaemic), prevention (primary vs. secondary) and device type (CRT-P vs. CRT-D)

Stratifying the comparison of Rh-negative vs. Rh-positive individuals by disease aetiology (ischemic vs. non-ischaemic) we observed no significant interaction as the point estimate was comparable for both groups: Ischemic HR 0.75 [0.47–1.19], p = 0.222 vs. Non-Ischemic: HR 0.61 [0.35–1.06], p = 0.080 (Supplementary Fig. 1). Similarly, no significant interaction was observed for device type (CRT-P: HR 0.65 [0.31–1.37], p = 0.255 vs. CRT-D: HR 0.68 [0.45–1.03], p = 0.070) or prevention (Primary prevention: HR 0.68 [0.46–1.01], p = 0.055 vs. Secondary prevention: HR 0.66 [0.26–1.69], p = 0.385).

### Associations of RHD & RHCE with other disease traits

Assessment of data from the GWAS-Catalog revealed 60 associations reported in the literature with RHCE (Supplementary Table 2) and 19 associations with RHD (Supplementary Table 3). For the RHD gene, 5 variants of interest were identified and the reported traits were related to platelets (plateletcrit, platelet count, and platelet distribution width), erythrocytes (mean corpuscular volume, mean corpuscular haemoglobin concentration, mean reticulocyte volume, and red cell distribution width), lipids (Omega-6 fatty acid and linoleic acid levels, phospholipid levels in medium VLDL, concentration of medium VLDL particles, cholesterol levels in small LDL particles, total lipid levels and cholesterol levels in small LDL) and others (pyruvate levels and mitochondrial DNA copy number).

The RHCE gene had 16 variants of interest and the reported traits were related to platelets (platelet count, plateletcrit and platelet distribution width), erythrocytes (mean corpuscular haemoglobin concentration, red cell distribution width, reticulocyte count, reticulocyte fraction of red cells, and mean reticulocyte volume), lipids (total cholesterol levels, LDL cholesterol levels, concentration of LDL particles, total cholesterol minus HDL-C levels, apolipoprotein B levels, cholesterol levels in medium and very small VLDL, cholesterol, phospholipid and total lipid levels in IDL, concentration of IDL particles) as well, among others (blood protein levels, glycated haemoglobin levels, direct bilirubin levels, serum levels of protein ICAM4, alopecia androgenetica, and heel bone mineral density). Genes in the closest proximity (RSRP1, TMEM50A, SYF2 and MACO1, all in 1p36.11—Supplementary Fig. 2) had similar associations in GWAS and no additional associations (e.g., with heart failure or cardiac disease) were identified.

Both RHD and RHCE were identified as druggable in DGIdb. Four drugs currently under development, all monoclonal antibodies, were identified targeting RHD: Rozrolimupab, Roledumab, Atorolimumab, and Morolimumab. Rozrolimupab and Roledumab have reached the phase 2 clinical stage, whilst the latter are phase 1 agents, and the planned clinical indications are treatment of autoimmune thrombocytopenic purpura and prevention of haemolytic disease of the newborn. Rozrolimupab, Roledumab, and Morolimumab are listed as inhibitors, and Atorolimumab is listed as “other”. There are no available drugs or drugs under development targeting RHCE.

## Discussion

In our study, we found a Rh-negative blood group to be an independent predictor of improved survival in HF patients implanted with CRT. To the best of our knowledge, this is the first time that this association has been reported.

The Rhesus status of a patient is defined by the presence of Rh factor, which is an inherited transmembrane protein located on the surface of red blood cells and found at 1p34.3-1p36.1^30^. Previous studies have identified an association between 1p34.3^18–20^ and 1p36.11^17^ with CAD, suggesting a genetic link between Rhesus status and cardiovascular disease. Our analysis of the GWAS-catalog provides potential mechanistic support for our observations in this cohort of patients with CRT. Interestingly, we did not observe an interaction of survival and aetiology which could be supported by the known association of Rhesus status and lipid levels. This suggests that the underlying mechanism may be more complex, and not explained solely by the observed association with lipid and glycated haemoglobin levels. Other possible affected pathways are those related to erythrocyte and platelet function. Also, we cannot rule out the possibility for linkage disequilibrium and of polymorphisms in the proximity of the Rhesus locus which may associate more frequently with negative Rhesus status and be the drivers of the observed survival benefit. Importantly, as the association seems to be independent of ethiology (ischemic vs. non-ischemic), it is possible that the causal underlying pathway is independent of atherosclerotic and thrombotic risk factors, which would likely manifest only through ischemic cardiomyopathy. Overall, our results are hypothesis-generating and suggest possible pathways worth exploring in the HF population. Furthermore, further assessment of a potential cardiac signal in the four identified drugs currently under development may be warranted. If our observations on the association of Rhesus status with prognosis are confirmed, and if drugs targeting RHD lead to a better survival depending on Rhesus status, our findings could align with the paradigm of precision medicine and genotype-guided treatment.

Despite evidence suggesting ABO blood group associates with incident myocardial infarction and HF, with a higher risk for non-O blood group patients^12^, demonstration of the impact of ABO blood group on prognosis is still scarce. Vaidya et al. have previously reported a higher rate of adverse events in O blood group patients irrespective of ventricular assist device use^31^. Gotsman et al. showed a non-O blood group to be an independent predictor of reduced survival in patients with non-ischaemic cardiomyopathy, but not in patients with ischaemic disease^24^. We could not reproduce this observation in our population of HF patients who received CRT. Interestingly, Gotsman et al. in a HF population comprising individuals with reduced and preserved ejection fraction HF observed that Rh-negative status was not associated with survival (HR 0.96 [0.68–1.35]). However, further sub-group analyses among the 55% of patients with data on Rhesus status (no information on CRT uptake), followed for a period of 4 years, showed improved survival in Rh-positive individuals with ischaemic cardiomyopathy. Interestingly, improved survival was observed for Rh-negative individuals with non-ischaemic cardiomyopathy during the initial 2 years of follow-up, with curves on time-to-event analysis starting to converge after that and finally overlapping at 3 years^24^. This abrupt change in slope violates the hazards assumptions for the Cox regression model (i.e., survival curves for different strata must have hazard functions that are proportional over the time), precluding meaningful interpretation of the data. Furthermore, the results of this study cannot be compared to our study due to pronounced differences in the cohorts, with the Israeli cohort being 12 years older in average, having more women, different ethnic composition, less patients with AF, and more than half of patients with > 50% LVEF.

Rhesus disease (haemolytic disease of the foetus and newborn) is a condition that develops when a Rh-negative woman conceives a Rh-positive fetus. It is caused by the presence of anti-Rhesus D IgG immunoglobulins that cross the placenta leading to haemolytic anaemia and several other complications, namely HF. Current knowledge is that to be high output HF occurs because of anaemia^32^. However, further insight into this matter and more detailed clarification of the mechanisms leading to HF may be required.

Currently available open-access bioinformatics resources like GWAS catalogue and OpenTargets constitute an important source for testing hypothesis and identifying possible mechanistic links or reasoning following findings from observational studies. As observed in our investigation, these resources are of interest when an association of a gene with an outcome is described as they allow us to understand the potential mechanistic links and whether the affected pathway can be modified by drugs, and hence constitute a druggable target for future use in clinical practice. On the other hand, attention is also required when drugs target an associated pathway as there is the potential to lead to that outcome as a possible side-effect, if the pathway is stimulated in the opposite direction.

### Limitations

We acknowledge that the present research has several limitations, including those that are inherent of an observational and retrospective study, including a relatively small number of patients, single centre-study design resulting in potential selection bias and inadvertently affecting the generalisability of our findings, and a lack of comparator group which would help to provide a more comprehensive understanding of this topic. A significant proportion of patients were referred from other centres with no echocardiographic information on LV dimensions being made available to us. Despite studies suggesting that LV diameter can have an important prognostic role in patients implanted with CRTs according to current guideline criteria^33,34^, a recent systematic review has identified LVEF as the main echocardiographic predictor that is present across HF risk models^35^.

In addition, as these data were obtained from a single UK centre our results cannot confirm whether the blood group of a patient predicts response to CRT as echo parameters were not assessed. We also did not have a group of patients without CRT to compare against. If patients without CRT and Rh-negative demonstrate improved clinical outcomes, this may indicate that Rh-negative is a predictor of improved outcomes in HF patients in general. If not, this may indicate that Rh-negative could represent a predictor of clinical CRT response. One of the inherent limitations of our study is the imbalance between the number of Rh-positive and Rh-negative individuals in the study cohort. As is commonly observed, the Rh-positive blood type prevails in most populations. The dominance of this poses a natural limitation, regardless of sample size, and our data aligns with the known prevalence for Rh-negative status in the population^36^. Another important limitation to acknowledge is the absence of a validation cohort. Although there are a lack of such studies within the HF population, in recent years the SARS-CoV-2 pandemic and the associated surge in research is an important source of knowledge as individuals with cardiovascular disease were a higher risk group susceptible to a more severe clinical course with increased mortality^37,38^. It is therefore interesting to note that Rh-negative individuals had less severe infection (e.g., need for intubation) and better survival when dealing with SARS-CoV-2 infection. Even though this has been corroborated by multiple publications^36,39,40^, it remains yet to be explained if the adverse outcome in Rh-positive individuals is driven by a higher prevalence of cardiovascular disease or other yet unknown factors. Finally, a small minority of patients had QRS < 130 ms and were implanted with CRTs based on practice in the 2000s which assumed a benefit in patients with narrower QRS complexes and presence of mechanical dyssynchrony^41^. Subsequent randomised controlled trials showed a lack of benefit of CRTs in the narrow QRS population^42,43^.

## Conclusion

Our results show that Rh-negative blood group is an independent predictor of improved outcomes in patients with HF who had CRT implantation. These findings warrant replication in other cohorts and have the potential to improve genetic risk prediction and the identification of novel drug targets for HF treatment.

### Supplementary Information

Supplementary Information.

## Data Availability

The datasets generated during and/or analysed during the current study are available from the corresponding author on reasonable request.

## References

[1] Bragazzi NL, Zhong W, Shu J, Abu Much A, Lotan D, Grupper A (2021). Burden of heart failure and underlying causes in 195 countries and territories from 1990 to 2017. Eur. J. Prev. Cardiol..

[2] Tsao CW, Aday AW, Almarzooq ZI, Alonso A, Beaton AZ, Bittencourt MS (2022). Heart disease and stroke statistics-2022 update: A report from the American heart association. Circulation..

[3] Bozkurt B, Hershberger RE, Butler J, Grady KL, Heidenreich PA, Isler ML (2021). 2021 ACC/AHA key data elements and definitions for heart failure: A report of the American College of Cardiology/American Heart Association Task force on clinical data standards (writing committee to develop clinical data standards for heart failure). Circ. Cardiovasc. Qual. Outcomes..

[4] Daubert C, Behar N, Martins RP, Mabo P, Leclercq C (2017). Avoiding non-responders to cardiac resynchronization therapy: A practical guide. Eur. Heart J..

[5] Schiavone M, Arosio R, Valenza S, Ruggiero D, Mitacchione G, Lombardi L (2023). Cardiac resynchronization therapy: Present and future. Eur. Heart J. Suppl..

[6] Providencia R, Marijon E, Barra S, Reitan C, Breitenstein A, Defaye P (2018). Usefulness of a clinical risk score to predict the response to cardiac resynchronization therapy. Int. J. Cardiol..

[7] Papageorgiou N, Providencia R, Lambiase PD, Tousoulis D, Lloyd G, Bhattacharyya S (2018). Does presence of left ventricular contractile reserve improve response to cardiac resynchronization therapy? An updated meta-analysis. Int. J. Cardiol..

[8] Ioannou A, Papageorgiou N, Barber H, Falconer D, Barra S, Babu G (2017). Impact of an age-adjusted co-morbidity index on survival of patients with heart failure implanted with cardiac resynchronization therapy devices. Am. J. Cardiol..

[9] Papageorgiou N, Falconer D, Ioannou A, Wongwarawipat T, Barra S, Tousoulis D (2019). Full blood count as potential predictor of outcomes in patients undergoing cardiac resynchronization therapy. Sci. Rep..

[10] Roselli C, Rienstra M, Ellinor PT (2020). Genetics of atrial fibrillation in 2020: GWAS, genome sequencing, polygenic risk, and beyond. Circ. Res..

[11] Shah S, Henry A, Roselli C, Lin H, Sveinbjornsson G, Fatemifar G (2020). Genome-wide association and Mendelian randomisation analysis provide insights into the pathogenesis of heart failure. Nat. Commun..

[12] Groot HE, Villegas Sierra LE, Said MA, Lipsic E, Karper JC, van der Harst P (2020). Genetically determined ABO blood group and its associations with health and disease. Arterioscler. Thromb. Vasc. Biol..

[13] Chung CM, Wang RY, Chen JW, Fann CS, Leu HB, Ho HY (2010). A genome-wide association study identifies new loci for ACE activity: Potential implications for response to ACE inhibitor. Pharmacogenom. J..

[14] Kiechl S, Pare G, Barbalic M, Qi L, Dupuis J, Dehghan A (2011). Association of variation at the ABO locus with circulating levels of soluble intercellular adhesion molecule-1, soluble P-selectin, and soluble E-selectin: A meta-analysis. Circ. Cardiovasc. Genet..

[15] Wu O, Bayoumi N, Vickers MA, Clark P (2008). ABO(H) blood groups and vascular disease: A systematic review and meta-analysis. J. Thromb. Haemost..

[16] Teslovich TM, Musunuru K, Smith AV, Edmondson AC, Stylianou IM, Koseki M (2010). Biological, clinical and population relevance of 95 loci for blood lipids. Nature..

[17] Hartiala JA, Han Y, Jia Q, Hilser JR, Huang P, Gukasyan J (2021). Genome-wide analysis identifies novel susceptibility loci for myocardial infarction. Eur. Heart J..

[18] Wang Q, Rao S, Shen GQ, Li L, Moliterno DJ, Newby LK (2004). Premature myocardial infarction novel susceptibility locus on chromosome 1P34-36 identified by genomewide linkage analysis. Am. J. Hum. Genet..

[19] Guo Y, Wang F, Li L, Gao H, Arckacki S, Wang IZ (2017). Genome-wide linkage analysis of large multiple multigenerational families identifies novel genetic loci for coronary artery disease. Sci. Rep..

[20] van der Harst P, Verweij N (2018). Identification of 64 novel genetic loci provides an expanded view on the genetic architecture of coronary artery disease. Circ. Res..

[21] (NICOR) NIfCOR. *National Clinical Audit on Device Implantation*. (2024).

[22] Ponikowski P, Voors AA, Anker SD, Bueno H, Cleland JGF, Coats AJS (2016). 2016 ESC Guidelines for the diagnosis and treatment of acute and chronic heart failure: The task force for the diagnosis and treatment of acute and chronic heart failure of the European Society of Cardiology (ESC)Developed with the special contribution of the Heart Failure Association (HFA) of the ESC. Eur. Heart J..

[23] Dahlen T, Clements M, Zhao J, Olsson ML, Edgren G (2021). An agnostic study of associations between ABO and RhD blood group and phenome-wide disease risk. Elife..

[24] Gotsman I, Keren A, Zwas DR, Lotan C, Admon D (2018). Clinical impact of ABO and Rhesus D blood type groups in patients with chronic heart failure. Am. J. Cardiol..

[25] Huang C, Chen Y, Reid M, Ghosh S (1996). Genetic recombination at the human RH locus: A family study of the red-cell Evans phenotype reveals a transfer of exons 2–6 from the RHD to the RHCE gene. Am. J. Hum. Genet..

[26] NHGRI-EBI Catalog of human genome-wide association studies (GWAS-Catalog). https://www.ebi.ac.uk/gwas/home. Accessed 15 Aug 2022.

[27] Drug Gene Interaction Database (DGIdb). https://dgidb.org/. Accessed 22 Aug 2022.

[28] Open Targets. https://www.opentargets.org/. Accessed 22 Aug 2022.

[29] ChEMBL. https://www.ebi.ac.uk/chembl/. Accessed 22 Aug 2022.

[30] Cherif-Zahar B, Mattei MG, Le Van KC, Bailly P, Cartron JP, Colin Y (1991). Localization of the human Rh blood group gene structure to chromosome region 1p34.3–1p36.1 by in situ hybridization. Hum. Genet..

[31] Vaidya GN, Trivedi J, Vijayakrishnan R, Fraser GE, Slaughter MS, Birks E (2020). Effect of blood group on heart transplant waitlist mortality in the ventricular assist device era. ASAIO J..

[32] Baldwin C, Pandey J, Olarewaju O (2022). Haemolytic Anaemia.

[33] Rinkuniene D, Bucyte S, Ceseviciute K, Abramavicius S, Baronaite-Dudoniene K, Laukaitiene J (2014). Predictors of positive response to cardiac resynchronization therapy. BMC Cardiovasc. Disord..

[34] Diaz-Infante E, Mont L, Leal J, Garcia-Bolao I, Fernandez-Lozano I, Hernandez-Madrid A (2005). Predictors of lack of response to resynchronization therapy. Am. J. Cardiol..

[35] Di Tanna GL, Wirtz H, Burrows KL, Globe G (2020). Evaluating risk prediction models for adults with heart failure: A systematic literature review. PLoS ONE..

[36] Anderson JL, May HT, Knight S, Bair TL, Horne BD, Knowlton KU (2022). Association of Rhesus factor blood type with risk of SARS-CoV-2 infection and COVID-19 severity. Br. J. Haematol..

[37] Williamson EJ, Walker AJ, Bhaskaran K, Bacon S, Bates C, Morton CE (2020). Factors associated with COVID-19-related death using OpenSAFELY. Nature..

[38] Strongman H, Carreira H, De Stavola BL, Bhaskaran K, Leon DA (2022). Factors associated with excess all-cause mortality in the first wave of the COVID-19 pandemic in the UK: A time series analysis using the Clinical Practice Research Datalink. PLoS Med..

[39] Ray JG, Schull MJ, Vermeulen MJ, Park AL (2021). Association between ABO and Rh blood groups and SARS-CoV-2 infection or severe COVID-19 illness: A population-based cohort study. Ann. Intern. Med..

[40] Zietz M, Zucker J, Tatonetti NP (2020). Associations between blood type and COVID-19 infection, intubation, and death. Nat. Commun..

[41] Cazeau SJ, Daubert JC, Tavazzi L, Frohlig G, Paul V (2008). Responders to cardiac resynchronization therapy with narrow or intermediate QRS complexes identified by simple echocardiographic indices of dyssynchrony: The DESIRE study. Eur. J. Heart Fail..

[42] Chung ES, Leon AR, Tavazzi L, Sun JP, Nihoyannopoulos P, Merlino J (2008). Results of the predictors of response to CRT (PROSPECT) trial. Circulation..

[43] Ruschitzka F, Abraham WT, Singh JP, Bax JJ, Borer JS, Brugada J (2013). Cardiac-resynchronization therapy in heart failure with a narrow QRS complex. N. Engl. J. Med..

