# Supplementary material for: Blood groups and Rhesus status as potential predictors of outcomes in patients with cardiac resynchronisation therapy
Source: Sci Rep. 2024 Apr 10;14:8371. doi: 10.1038/s41598-024-58747-8 (PMC11006901; doi:10.1038/s41598-024-58747-8)

**Supplementary Table 1.** Multivariate Cox Regression models for the role of Rh-negative blood group as an independent predictor of survival.

|  | **Multivariate Cox Regression**  **(Method: Enter)** | | | **Multivariate Cox Regression**  **(Backward LR)** | | |
| --- | --- | --- | --- | --- | --- | --- |
| Predictor | HR | 95% CI | P | HR | 95% CI | P |
| Age (per year) | 1.01 | 1.00-1.02 | 0.102 | 1.01 | 1.00-1.02 | 0.068 |
| Women | 0.77 | 0.57-1.03 | 0.074 | 0.76 | 0.57-1.03 | 0.075 |
| BMI (per Kg/m^2^) | 0.98 | 0.96-1.00 | 0.035 | 0.98 | 0.97-1.00 | 0.038 |
| Ischaemic CM | 1.03 | 0.78-1.36 | 0.842 | - | - | - |
| LVEF (%) | 0.99 | 0.98-1.00 | 0.044 | 0.99 | 0.97-1.00 | 0.025 |
| QRS ≥150ms | 0.55 | 0.42-0.71 | <0.001 | 0.54 | 0.42-0.71 | <0.001 |
| NYHA class (per class) | 1.76 | 1.42-2.17 | <0.001 | 1.76 | 1.42-2.17 | - |
| AF | 1.22 | 0.96-1.56 | 0.111 | 1.23 | 0.96-1.57 | 0.097 |
| COPD | 1.08 | 0.73-1.59 | 0.703 | - | - | - |
| Previous stroke | 1.17 | 0.77-1.78 | 0.457 | - | - | - |
| eGFR ≥60ml/min | 0.66 | 0.50-0.88 | 0.004 | 0.66 | 0.50-0.86 | 0.003 |
| Rhesus neg (-) | 1.51 | 1.04-2.18 | 0.028 | 1.52 | 1.06-2.20 | 0.024 |
| Non-O group | 0.96 | 0.76-1.23 | 0.762 | - | - | - |

**Supplementary Table 2.** Association of RHCE with other disease traits.

| **Mapped gene** | **Variant and risk allele** | **Beta** | **P-value** | **Reported trait** | **Location** |
| --- | --- | --- | --- | --- | --- |
| [RHCE](https://www.ebi.ac.uk/gwas/genes/RHCE) | [rs1293259-A](https://www.ebi.ac.uk/gwas/variants/rs1293259) | 0.0361 unit increase | 1 x 10^-25^ | Glycated hemoglobin levels | 1:25369344 |
| [RHCE](https://www.ebi.ac.uk/gwas/genes/RHCE) | [rs1293259-A](https://www.ebi.ac.uk/gwas/variants/rs1293259) | 0.024 unit increase | 1 x 10^-12^ | Low density lipoprotein cholesterol levels | 1:25369344 |
| [RHCE](https://www.ebi.ac.uk/gwas/genes/RHCE) | [rs1293259-A](https://www.ebi.ac.uk/gwas/variants/rs1293259) | 0.0272 unit increase | 7 x 10^-16^ | Apolipoprotein B levels | 1:25369344 |
| [RHCE](https://www.ebi.ac.uk/gwas/genes/RHCE) | [rs1293259-A](https://www.ebi.ac.uk/gwas/variants/rs1293259) | 0.0224 unit increase | 3 x 10^-11^ | Total cholesterol levels | 1:25369344 |
| [RHCE](https://www.ebi.ac.uk/gwas/genes/RHCE) | [rs1293259-A](https://www.ebi.ac.uk/gwas/variants/rs1293259) | 0.0231 unit decrease | 2 x 10^-10^ | Direct bilirubin levels | 1:25369344 |
| [RHCE](https://www.ebi.ac.uk/gwas/genes/RHCE) | [rs1293259-A](https://www.ebi.ac.uk/gwas/variants/rs1293259) | 0.415041 unit increase | 4 x 10^-62^ | Serum levels of protein ICAM4 | 1:25369344 |
| [RHCE](https://www.ebi.ac.uk/gwas/genes/RHCE) | [rs644592-T](https://www.ebi.ac.uk/gwas/variants/rs644592) | 0.026704641 unit decrease | 9 x 10^-18^ | High light scatter reticulocyte count | 1:25376665 |
| [RHCE](https://www.ebi.ac.uk/gwas/genes/RHCE) | [rs644592-T](https://www.ebi.ac.uk/gwas/variants/rs644592) | 0.039737374 unit decrease | 6 x 10^-36^ | Reticulocyte count | 1:25376665 |
| [RHCE](https://www.ebi.ac.uk/gwas/genes/RHCE) | [rs644592-T](https://www.ebi.ac.uk/gwas/variants/rs644592) | 0.038129624 unit decrease | 3 x 10^-33^ | Reticulocyte fraction of red cells | 1:25376665 |
| [RHCE](https://www.ebi.ac.uk/gwas/genes/RHCE) | [rs621189-T](https://www.ebi.ac.uk/gwas/variants/rs621189) | 0.1829599 unit decrease | 3 x 10^-11^ | Mean corpuscular hemoglobin concentration | 1:25383919 |
| [RHCE](https://www.ebi.ac.uk/gwas/genes/RHCE) | [rs1293261-A](https://www.ebi.ac.uk/gwas/variants/rs1293261) | 0.0262636 unit decrease | 2 x 10^-10^ | Cholesterol levels in medium VLDL | 1:25386743 |
| [RHCE](https://www.ebi.ac.uk/gwas/genes/RHCE) | [rs1293261-A](https://www.ebi.ac.uk/gwas/variants/rs1293261) | 0.0266463 unit decrease | 7 x 10^-11^ | Cholesteryl ester levels in medium VLDL | 1:25386743 |
| [RHCE](https://www.ebi.ac.uk/gwas/genes/RHCE) | [rs1293261-A](https://www.ebi.ac.uk/gwas/variants/rs1293261) | 0.0240683 unit decrease | 5 x 10^-9^ | Free cholesterol levels in medium VLDL | 1:25386743 |
| [RHCE](https://www.ebi.ac.uk/gwas/genes/RHCE) | [rs1293261-A](https://www.ebi.ac.uk/gwas/variants/rs1293261) | 0.0226504 unit decrease | 4 x 10^-8^ | Total cholesterol minus HDL-C levels | 1:25386743 |
| [RHCE](https://www.ebi.ac.uk/gwas/genes/RHCE) | [rs1293261-A](https://www.ebi.ac.uk/gwas/variants/rs1293261) | 0.023626 unit decrease | 8 x 10^-9^ | Remnant cholesterol (non-HDL, non-LDL -cholesterol) | 1:25386743 |
| [RHCE](https://www.ebi.ac.uk/gwas/genes/RHCE) | [rs1293261-A](https://www.ebi.ac.uk/gwas/variants/rs1293261) | 0.0227603 unit decrease | 4 x 10^-8^ | Cholesteryl ester levels in VLDL | 1:25386743 |
| [RHCE](https://www.ebi.ac.uk/gwas/genes/RHCE) | [rs1293261-A](https://www.ebi.ac.uk/gwas/variants/rs1293261) | 0.0230029 unit decrease | 8 x 10^-9^ | Cholesteryl ester levels in very small VLDL | 1:25386743 |
| [RHCE](https://www.ebi.ac.uk/gwas/genes/RHCE) | [rs1293261-A](https://www.ebi.ac.uk/gwas/variants/rs1293261) | 0.0243776 unit decrease | 3 x 10^-9^ | Apolipoprotein B levels | 1:25386743 |
| [RHCE](https://www.ebi.ac.uk/gwas/genes/RHCE) | [rs1293261-A](https://www.ebi.ac.uk/gwas/variants/rs1293261) | 0.0227195 unit decrease | 2 x 10^-8^ | Free cholesterol levels in very small VLDL | 1:25386743 |
| [RHCE](https://www.ebi.ac.uk/gwas/genes/RHCE) | [rs1293261-A](https://www.ebi.ac.uk/gwas/variants/rs1293261) | 0.0231386 unit decrease | 7 x 10^-9^ | Cholesterol levels in very small VLDL | 1:25386743 |
| [RHCE](https://www.ebi.ac.uk/gwas/genes/RHCE) | [rs1293261-A](https://www.ebi.ac.uk/gwas/variants/rs1293261) | 0.0230189 unit decrease | 1 x 10^-8^ | Total free cholesterol levels | 1:25386743 |
| [RHCE](https://www.ebi.ac.uk/gwas/genes/RHCE) | [rs1293261-A](https://www.ebi.ac.uk/gwas/variants/rs1293261) | 0.0218899 unit decrease | 4 x 10^-8^ | Cholesterol levels in IDL | 1:25386743 |
| [RHCE](https://www.ebi.ac.uk/gwas/genes/RHCE) | [rs1293261-A](https://www.ebi.ac.uk/gwas/variants/rs1293261) | 0.0222132 unit decrease | 3 x 10^-8^ | Total lipid levels in IDL | 1:25386743 |
| [RHCE](https://www.ebi.ac.uk/gwas/genes/RHCE) | [rs1293261-A](https://www.ebi.ac.uk/gwas/variants/rs1293261) | 0.0227873 unit decrease | 3 x 10^-8^ | Concentration of IDL particles | 1:25386743 |
| [RHCE](https://www.ebi.ac.uk/gwas/genes/RHCE) | [rs1293261-A](https://www.ebi.ac.uk/gwas/variants/rs1293261) | 0.0234801 unit decrease | 4 x 10^-9^ | Phospholipid levels in IDL | 1:25386743 |
| [RHCE](https://www.ebi.ac.uk/gwas/genes/RHCE) | [rs1293261-A](https://www.ebi.ac.uk/gwas/variants/rs1293261) | 0.0245832 unit decrease | 3 x 10^-9^ | Concentration of LDL particles | 1:25386743 |
| [RHCE](https://www.ebi.ac.uk/gwas/genes/RHCE) | [rs1293261-A](https://www.ebi.ac.uk/gwas/variants/rs1293261) | 0.0231466 unit decrease | 2 x 10^-8^ | Free cholesterol levels in small VLDL | 1:25386743 |
| [RHCE](https://www.ebi.ac.uk/gwas/genes/RHCE) | [rs3079633-A](https://www.ebi.ac.uk/gwas/variants/rs3079633) | 0.62 unit decrease | 2 x 10^-160^ | Blood protein levels | 1:25386843 |
| [RHCE](https://www.ebi.ac.uk/gwas/genes/RHCE) | [rs3079633-A](https://www.ebi.ac.uk/gwas/variants/rs3079633) | 0.17 unit increase | 5 x 10^-12^ | Blood protein levels | 1:25386843 |
| [RHCE](https://www.ebi.ac.uk/gwas/genes/RHCE) | [rs35992941-A](https://www.ebi.ac.uk/gwas/variants/rs35992941) | 0.023165377 unit decrease | 4 x 10^-25^ | Mean corpuscular hemoglobin | 1:25386843 |
| [RHCE](https://www.ebi.ac.uk/gwas/genes/RHCE) | [rs760938-C](https://www.ebi.ac.uk/gwas/variants/rs760938) | 0.0113471 unit increase | 7 x 10^-10^ | Heel bone mineral density | 1:25389394 |
| [RHCE](https://www.ebi.ac.uk/gwas/genes/RHCE) | [rs679429-A](https://www.ebi.ac.uk/gwas/variants/rs679429) | 0.0206062 unit increase | 2 x 10^-11^ | Plateletcrit | 1:25393597 |
| [RHCE](https://www.ebi.ac.uk/gwas/genes/RHCE) | [rs376477778-G](https://www.ebi.ac.uk/gwas/variants/rs376477778) | 0.028651364 unit decrease | 9 x 10^-15^ | Reticulocyte count | 1:25398010 |
| [RHCE](https://www.ebi.ac.uk/gwas/genes/RHCE) | [rs376477778-G](https://www.ebi.ac.uk/gwas/variants/rs376477778) | 0.028207112 unit decrease | 2 x 10^-14^ | Reticulocyte fraction of red cells | 1:25398010 |
| [RHCE](https://www.ebi.ac.uk/gwas/genes/RHCE) | [rs61777615-A](https://www.ebi.ac.uk/gwas/variants/rs61777615) | 0.04409033 unit decrease | 6 x 10^-34^ | Red cell distribution width | 1:25405596 |
| [RHCE](https://www.ebi.ac.uk/gwas/genes/RHCE) | [rs61777615-A](https://www.ebi.ac.uk/gwas/variants/rs61777615) | 0.0399529 unit decrease | 3 x 10^-24^ | Red cell distribution width | 1:25405596 |
| [RHCE](https://www.ebi.ac.uk/gwas/genes/RHCE) | [rs28513325-A](https://www.ebi.ac.uk/gwas/variants/rs28513325) | 0.09227352 unit decrease | 1 x 10^-13^ | Reticulocyte count | 1:25406057 |
| [RHCE](https://www.ebi.ac.uk/gwas/genes/RHCE) | [rs28513325-A](https://www.ebi.ac.uk/gwas/variants/rs28513325) | 0.08774246 unit decrease | 2 x 10^-12^ | Reticulocyte fraction of red cells | 1:25406057 |
| [RHCE](https://www.ebi.ac.uk/gwas/genes/RHCE) | [rs28513325-A](https://www.ebi.ac.uk/gwas/variants/rs28513325) | 0.052976456 unit decrease | 5 x 10^-15^ | High light scatter reticulocyte count | 1:25406057 |
| [RHCE](https://www.ebi.ac.uk/gwas/genes/RHCE) | [rs28513325-A](https://www.ebi.ac.uk/gwas/variants/rs28513325) | 0.051504366 unit decrease | 3 x 10^-14^ | High light scatter reticulocyte percentage of red cells | 1:25406057 |
| [RHCE](https://www.ebi.ac.uk/gwas/genes/RHCE) | [rs28513325-A](https://www.ebi.ac.uk/gwas/variants/rs28513325) | 0.07650593 unit increase | 4 x 10^-23^ | Mean spheric corpuscular volume | 1:25406057 |
| [RHCE](https://www.ebi.ac.uk/gwas/genes/RHCE) | [rs28513325-A](https://www.ebi.ac.uk/gwas/variants/rs28513325) | 0.050950553 unit increase | 5 x 10^-11^ | Mean reticulocyte volume | 1:25406057 |
| [RHCE](https://www.ebi.ac.uk/gwas/genes/RHCE) | [rs111941366-T](https://www.ebi.ac.uk/gwas/variants/rs111941366) | - | 9 x 10^-81^ | Platelet count | 1:25409878 |
| [RHCE](https://www.ebi.ac.uk/gwas/genes/RHCE) | [rs111941366-T](https://www.ebi.ac.uk/gwas/variants/rs111941366) | 0.036054 SD unit decrease | 1 x 10^-84^ | Platelet count | 1:25409878 |
| [RHCE](https://www.ebi.ac.uk/gwas/genes/RHCE) | [rs111941366-T](https://www.ebi.ac.uk/gwas/variants/rs111941366) | 0.02634887 unit increase | 4 x 10^-13^ | Platelet distribution width | 1:25409878 |
| [RHCE](https://www.ebi.ac.uk/gwas/genes/RHCE) | [rs111941366-T](https://www.ebi.ac.uk/gwas/variants/rs111941366) | 0.04322901 unit decrease | 6 x 10^-32^ | Platelet count | 1:25409878 |
| [RHCE](https://www.ebi.ac.uk/gwas/genes/RHCE) | [rs78207142-T](https://www.ebi.ac.uk/gwas/variants/rs78207142) | 0.032565027 unit increase | 9 x 10^-17^ | Platelet count | 1:25416203 |
| [RHCE](https://www.ebi.ac.uk/gwas/genes/RHCE) | [rs2982360-T](https://www.ebi.ac.uk/gwas/variants/rs2982360) | 0.025852455 unit decrease | 2 x 10^-14^ | Mean spheric corpuscular volume | 1:25419154 |
| [RHCE](https://www.ebi.ac.uk/gwas/genes/RHCE) | [rs586178-G](https://www.ebi.ac.uk/gwas/variants/rs586178) | 0.025 unit decrease | 2 x 10^-6^ | Low density lipoprotein cholesterol levels | 1:25420739 |
| [RHCE](https://www.ebi.ac.uk/gwas/genes/RHCE) | [rs586178-G](https://www.ebi.ac.uk/gwas/variants/rs586178) | 0.026 unit decrease | 9 x 10^-8^ | Low density lipoprotein cholesterol levels | 1:25420739 |
| [RHCE](https://www.ebi.ac.uk/gwas/genes/RHCE) | [rs586178-G](https://www.ebi.ac.uk/gwas/variants/rs586178) | - | 5 x 10^-136^ | Red cell distribution width | 1:25420739 |
| [RHCE](https://www.ebi.ac.uk/gwas/genes/RHCE) | [rs586178-G](https://www.ebi.ac.uk/gwas/variants/rs586178) | 0.047846 SD unit decrease | 5 x 10^-139^ | Red cell distribution width | 1:25420739 |
| [RHCE](https://www.ebi.ac.uk/gwas/genes/RHCE) | [rs586178-G](https://www.ebi.ac.uk/gwas/variants/rs586178) | 0.026 unit decrease | 4 x 10^-7^ | Total cholesterol levels | 1:25420739 |
| [RHCE](https://www.ebi.ac.uk/gwas/genes/RHCE) | [rs586178-G](https://www.ebi.ac.uk/gwas/variants/rs586178) | 0.026 unit decrease | 6 x 10^-8^ | Total cholesterol levels | 1:25420739 |
| [RHCE](https://www.ebi.ac.uk/gwas/genes/RHCE) | [rs586178-C](https://www.ebi.ac.uk/gwas/variants/rs586178) | 0.037 s.d. increase | 4 x 10^-9^ | LDL cholesterol | 1:25420739 |
| [RHCE](https://www.ebi.ac.uk/gwas/genes/RHCE) | [rs586178-C](https://www.ebi.ac.uk/gwas/variants/rs586178) | 0.037 s.d. increase | 3 x 10^-9^ | Cholesterol, total | 1:25420739 |
| [RHCE](https://www.ebi.ac.uk/gwas/genes/RHCE) | [rs586178-?](https://www.ebi.ac.uk/gwas/variants/rs586178) | - | 3 x 10^-123^ | Red cell distribution width | 1:25420739 |
| [RHCE](https://www.ebi.ac.uk/gwas/genes/RHCE) | [rs586178-C](https://www.ebi.ac.uk/gwas/variants/rs586178) | 0.06871988 unit increase | 3 x 10^-102^ | Red cell distribution width | 1:25420739 |
| [RHCE](https://www.ebi.ac.uk/gwas/genes/RHCE) | [rs28695210-A](https://www.ebi.ac.uk/gwas/variants/rs28695210) | 0.0481 unit increase | 1 x 10^-8^ | Low density lipoprotein cholesterol levels | 1:25422778 |
| [RHCE](https://www.ebi.ac.uk/gwas/genes/RHCE) | [rs1293259-A](https://www.ebi.ac.uk/gwas/variants/rs1293259) | 0.418982 unit increase | 2 x 10^-63^ | Serum levels of protein ICAM4 | 1:25369344 |

**Supplementary Table 3.** Association of RHD with other disease traits.

| **Mapped gene** | **Variant and risk allele** | **Beta** | **P-value** | **Reported trait** | **Location** |
| --- | --- | --- | --- | --- | --- |
| RHD | [rs604183-A](https://www.ebi.ac.uk/gwas/variants/rs604183) | 0.0386853 unit decrease | 6 x 10-68 | Mean corpuscular volume | 1:25274256 |
| RHD | [rs604183-A](https://www.ebi.ac.uk/gwas/variants/rs604183) | 0.026953 unit increase | 5 x 10-45 | Mean corpuscular hemoglobin concentration | 1:25274256 |
| RHD | [rs604183-A](https://www.ebi.ac.uk/gwas/variants/rs604183) | 0.06587745 unit decrease | 7 x 10-94 | Mean spheric corpuscular volume | 1:25274256 |
| RHD | [rs604183-A](https://www.ebi.ac.uk/gwas/variants/rs604183) | 0.028446522 unit decrease | 6 x 10-27 | Platelet count | 1:25274256 |
| RHD | [rs604183-A](https://www.ebi.ac.uk/gwas/variants/rs604183) | 0.052279644 unit decrease | 5 x 10-107 | Mean reticulocyte volume | 1:25274256 |
| RHD | [rs604183-A](https://www.ebi.ac.uk/gwas/variants/rs604183) | 0.057684317 unit decrease | 1 x 10-84 | Plateletcrit | 1:25274256 |
| RHD | [rs604183-A](https://www.ebi.ac.uk/gwas/variants/rs604183) | 0.02699633 unit increase | 1 x 10-32 | Platelet distribution width | 1:25274256 |
| RHD | [rs604183-A](https://www.ebi.ac.uk/gwas/variants/rs604183) | 0.0334 unit decrease | 3 x 10-44 | Mean corpuscular volume | 1:25274256 |
| RHD | [rs604183-A](https://www.ebi.ac.uk/gwas/variants/rs604183) | 0.0358 unit decrease | 7 x 10-53 | Platelet count | 1:25274256 |
| RHD | [rs2986164-G](https://www.ebi.ac.uk/gwas/variants/rs2986164) | 0.0249355 unit increase | 3 x 10-8 | Phospholipid levels in medium VLDL | 1:25295800 |
| RHD | [rs2986164-G](https://www.ebi.ac.uk/gwas/variants/rs2986164) | 0.0245171 unit increase | 5 x 10-8 | Concentration of medium VLDL particles | 1:25295800 |
| RHD | [rs2986164-G](https://www.ebi.ac.uk/gwas/variants/rs2986164) | 0.0247173 unit increase | 5 x 10-8 | Cholesterol levels in small LDL | 1:25295800 |
| RHD | [rs2986164-G](https://www.ebi.ac.uk/gwas/variants/rs2986164) | 0.0247791 unit increase | 2 x 10-8 | Omega-6 fatty acid levels | 1:25295800 |
| RHD | [rs2986164-G](https://www.ebi.ac.uk/gwas/variants/rs2986164) | 0.0256176 unit increase | 1 x 10-8 | Total lipid levels in small LDL | 1:25295800 |
| RHD | [rs2986164-G](https://www.ebi.ac.uk/gwas/variants/rs2986164) | 0.0265969 unit increase | 4 x 10-9 | Concentration of small LDL particles | 1:25295800 |
| RHD | [rs2986164-G](https://www.ebi.ac.uk/gwas/variants/rs2986164) | 0.0262998 unit increase | 3 x 10-9 | Linoleic acid levels | 1:25295800 |
| RHD | [rs143670081-?](https://www.ebi.ac.uk/gwas/variants/rs143670081) | - | 4 x 10-28 | Red cell distribution width | 1:25295753 |
| RHD | [rs139898146-C](https://www.ebi.ac.uk/gwas/variants/rs139898146) | 0.0269081 unit increase | 1 x 10-10 | Pyruvate levels | 1:25274256 |
| RHD | [rs139898146-A](https://www.ebi.ac.uk/gwas/variants/rs139898146) | 0.0468 unit increase | 2 x 10-106 | Mitochondrial DNA copy number | 1:25274256 |

**Supplementary Figure 1.** Sub-analyses for the effect of Rhesus status by ischaemic vs. non-ischaemic groups.


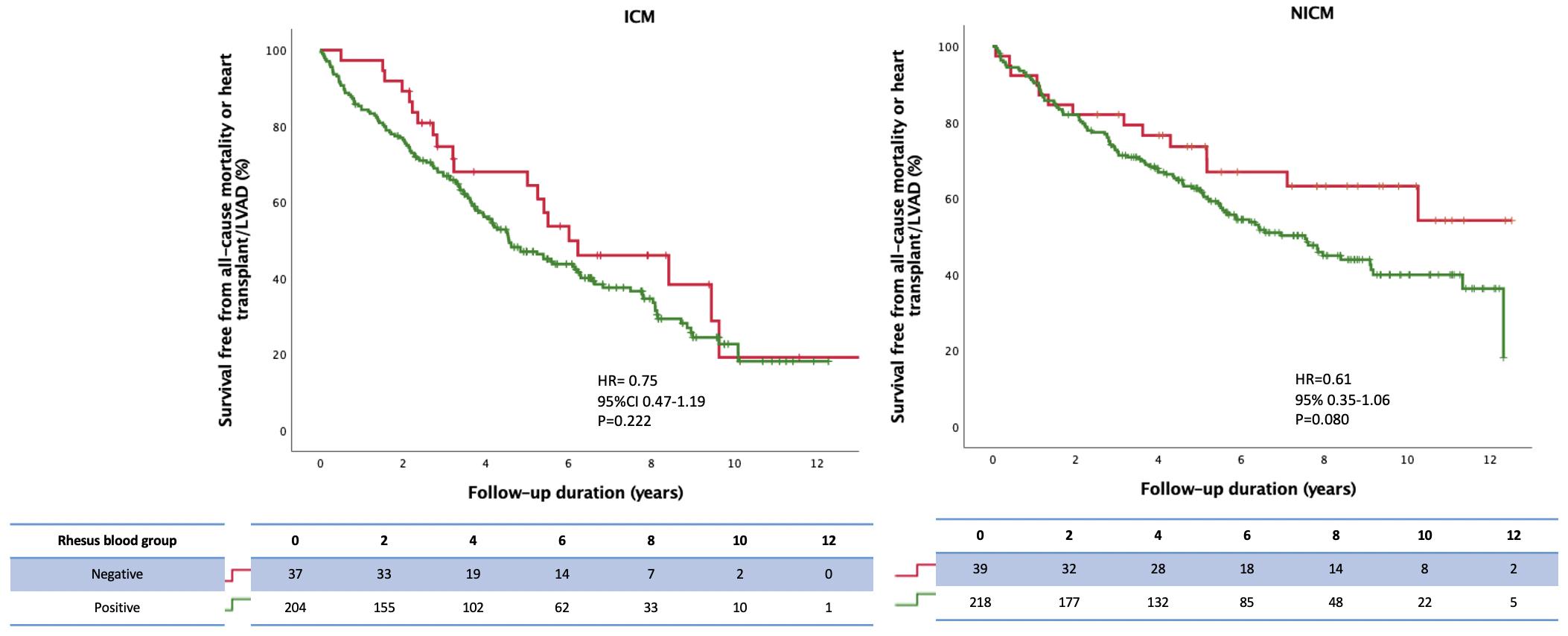


**Supplementary Figure 2.** Genes in the proximity of RHD and RHCE. Modified from Open Targets Genetics and covers Locus 1:24,301,419-26,301,419.


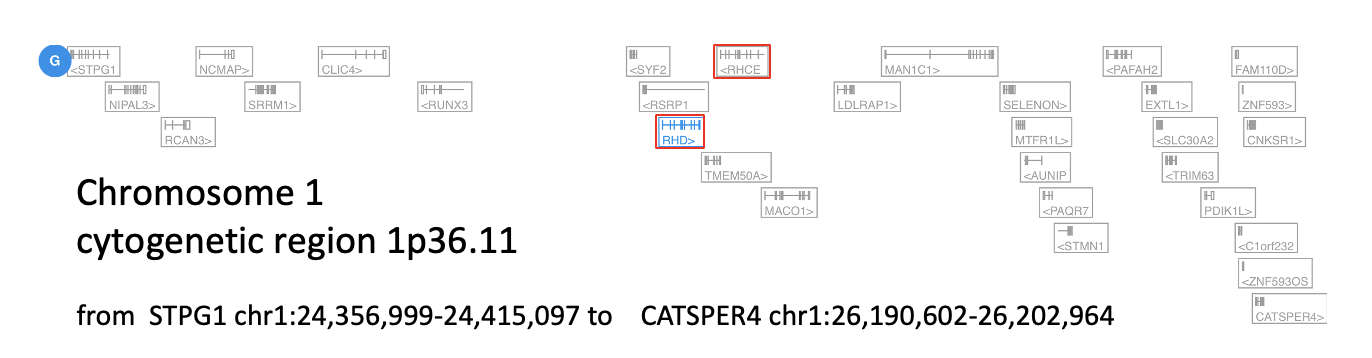

Supplement: Supplementary file 1 — Supplementary Information. [file 41598_2024_58747_MOESM1_ESM.docx]
